# Free-Space Nonlinear Beam Combining for High Intensity Projection

**DOI:** 10.1038/s41598-017-10565-x

**Published:** 2017-08-31

**Authors:** Shermineh Rostami Fairchild, Wiktor Walasik, Daniel Kepler, Matthieu Baudelet, Natalia M. Litchinitser, Martin Richardson

**Affiliations:** 1 Laser Plasma Laboratory, Townes Laser Institute, College of Optics and Photonics, University of Central Florida, Orlando, USA; 2 Department of Electrical Engineering, University at Buffalo, The State University of New York, Buffalo, New York, USA; 3 National Center for Forensic Science, Chemistry department, University of Central Florida, Orlando, USA

## Abstract

The controlled interaction of two high intensity beams opens new degrees of freedom for manipulating electromagnetic waves in air. The growing number of applications for laser filaments requires fine control of their formation and propagation. We demonstrate, experimentally and theoretically, that the attraction and fusion of two parallel ultrashort beams with initial powers below the critical value (70% *P*_critical_), in the regime where the non-linear optical characteristics of the medium become dominant, enable the eventual formation of a filament downstream. Filament formation is delayed to a predetermined distance in space, defined by the initial separation between the centroids, while still enabling filaments with controllable properties as if formed from a single above-critical power beam. This is confirmed by experimental and theoretical evidence of filament formation such as the individual beam profiles and the supercontinuum emission spectra associated with this interaction.

## Introduction

Filament generation in air is a nonlinear process requiring high-power ultrashort laser pulses (>3 GW for 800 nm)^1, 2^ to induce a dynamic balance between Kerr self-focusing and plasma defocusing. The unique properties of these self-channeled structures, such as diffraction-free propagation and the formation of plasma channels many times longer than the Rayleigh length, allow for the development of a wide variety of applications^3^. Arrays of filaments have been shown to enable various guided-wave structures in the free space for visible^4^, infrared^5^, radio and microwave^6–8^ frequency radiation, depending on their configuration. The generation of large arrays of filaments calls for phase manipulation of the laser pulses as well as precise engineering of intensity/phase distributions and the nonlinear beam interactions^8–14^.

Nearly two decades of experimental and theoretical studies of filamentation in air with near-infrared femtosecond laser pulses, have elucidated the principal physical processes involved^15–20^. Most studies have been performed with laser powers sufficient for the eventual formation of a filament, in air stabilized by partial ionization and the consequential impact of the liberated electrons on the beam propagation. The present study focuses on the processes involved in the initial Kerr-lens focusing of the beam, where its distribution changes progressively to the Townesian distribution characteristic of a single propagating filament. We consider the case for beams propagating in the nonlinear regime, but with powers below that for filamentation. The impetus for the present study is to understand how beams in this regime interact with one another when they are launched parallel and in close proximity. Early experimental studies examined the interaction between filaments, either in multi-filamenting beams^21–24^, between two parallel filaments^25–28^, or filaments with non-parallel propagation direction^29–31^. The separation between the intensity and/or phase perturbations within the beam spatial profile was shown to have an effect on the growth of multiple filaments, the diameter of the plasma channel, and the overall electron density^24^. The importance of the relative phase between propagating parallel beams has been recognized theoretically for a long time^32, 33^. Phase-controlled interactions between two or more filaments have been the subject of number of investigations^25–36^, including phase-engineered helical filaments^37^. All these cases were based on laser beams with initial powers greater than the critical value to ensure the interaction between already fully stabilized filaments.

Controlling the formation of filaments and managing the high intensity induced by the filamenting beams on projection optics, are still among the most significant fundamental and technological challenges. We propose a solution to these issues by exploiting nonlinear coherent combining of two beams with initial powers below the critical value, *P*_cr_, to create filaments. We examine in detail, both experimentally and theoretically, the interaction between two in-phase ultrashort beams, as their centroids are moved closer together. Our results demonstrate clear attraction and fusion of ultrashort beams, with powers below the critical value for filamentation, towards formation of a single filament. We believe this knowledge will have an impact on studies with arrays of multiple filaments, remote THz generation^38^, supercontinuum generation^39^, and applications of organized filament structures^8^.

**Figure 1:**
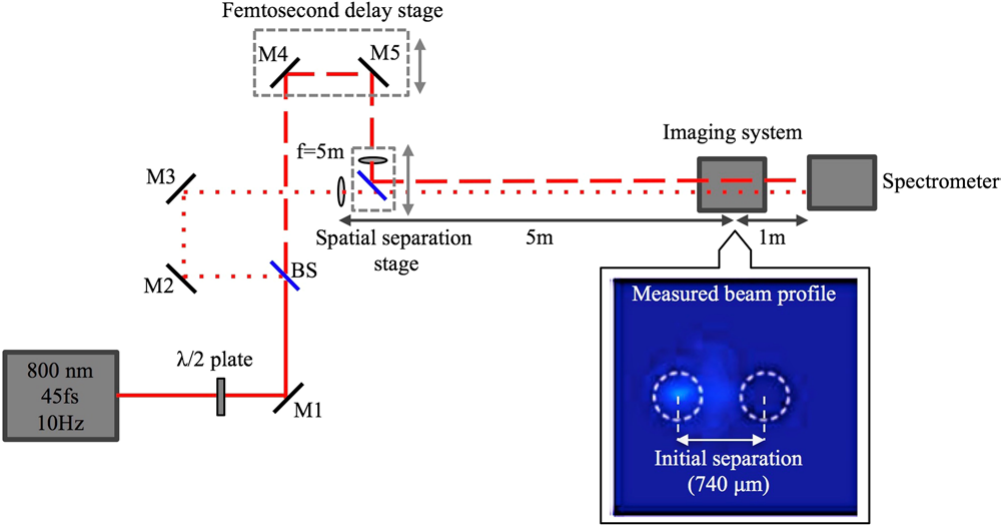
Experimental setup. The inset shows the beam profiles measured at the geometrical focus when the initial separation between the two beams was 740 μm.

**Figure 2:**
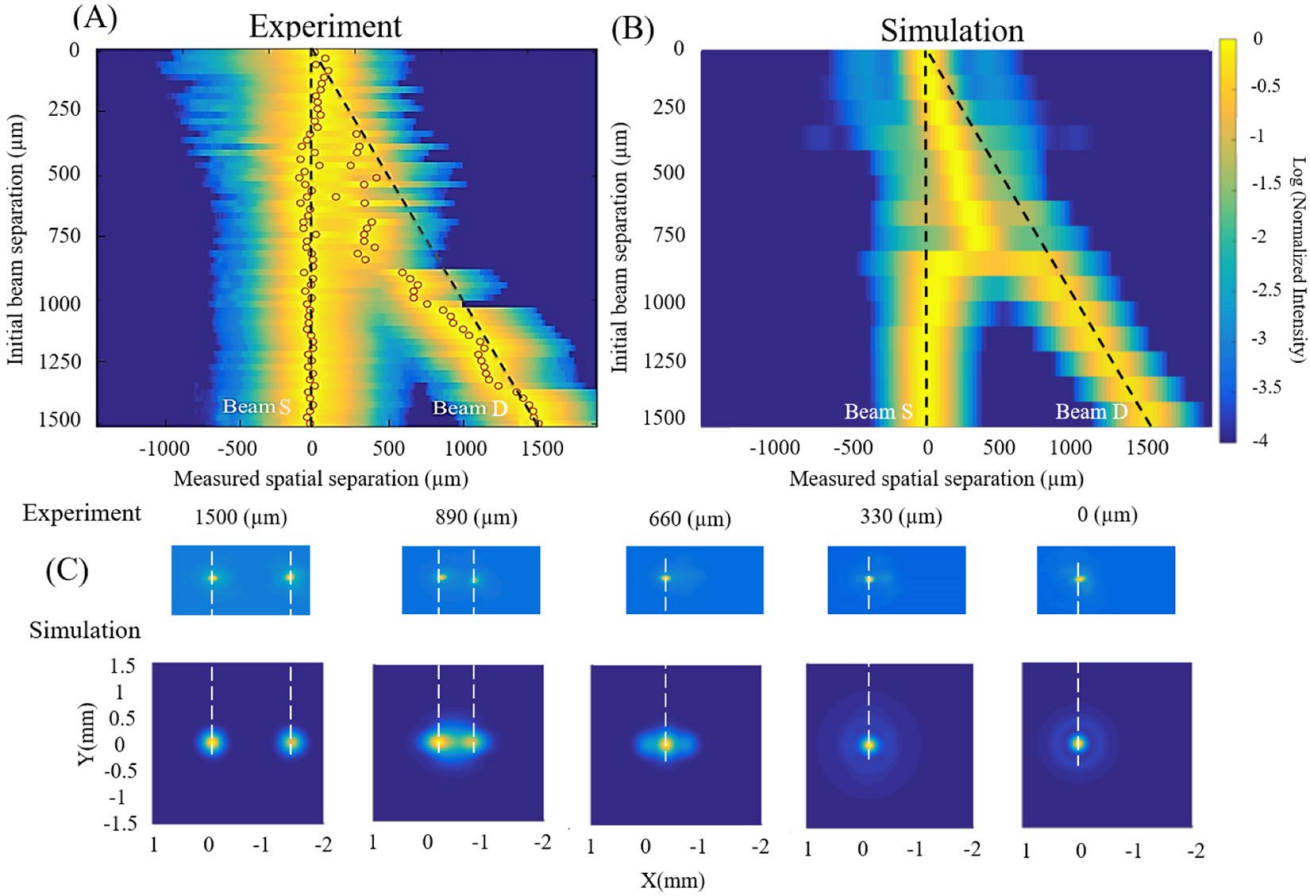
(**A**) Measured and (**B**) calculated normalized intensity profiles of the 2 beams in the transverse direction *x* at the geometrical focus (*z* = 5 m). Black dashed lines show the initial separation between the stationary (beam S) and dynamic (beam D) beams and red circles denote the local intensity maxima in the experimental profiles. (**C**) Experimental (top row) and simulated (bottom row) beam profiles in the transverse plane (*x*-*y*) for 1500, 890, 660, 330, and 0 μm initial separation. White dashed lines indicate the position of the local intensity maxima in the simulation results.

The experiment was conducted using the Multi-Terawatt Femtosecond Laser (MTFL)^40^ at the University of Central Florida, with an output at 10 Hz of 45 fs pulses (full width at half-maximum—FWHM) at 800 nm. The horizontally polarized laser pulses were rotated with a half-wave plate (λ/2) to allow for maximum transmission through the interferometer (Fig. 1). A beam splitter (BS) was then used to separate the beam between two arms. After travelling equal distances and passing through identical focusing lenses (*f* = 5 m, nonlinear focusing regime^41^), the two beams were recombined at the second beam splitter. The beam splitter and one of the lenses were mounted on a translation stage to enable fine control of the spatial separation of the centroids of the two beams, keeping the geometrical focus fixed. The relative phase between the two arms was controlled spatially and temporally between the beams to a resolution of 25.4 μm and ~7 fs, respectively. This was done to make sure the beams were in-phase at each separation, confirmed by their constructive interference. The two linearly polarized Gaussian beams, each with 4.25 mm diameter (FWHM) in the transverse plane, starting with 0.72*P*_cr_ and 0.68*P*_cr_ peak powers, were spatially brought into horizontal proximity to one another in steps of 25.4 μm and overlapped in time for each step. To reduce spatial positioning errors, one of the arms (beam S with 0.72P_cr_) was kept fixed and only the other arm (beam D with 0.68P_cr_) was moved relative to beam S (Fig. 2A).

The overall beam profile (Fig. 2A) was measured at the geometrical focus, using a series of grazing incidence uncoated fused silica wedges as an attenuator. The large angle of incidence relative to the surface normal prevents surface damage from the high intensity filaments. The low reflectivity attenuates the beam intensity by a factor of 10^7^ allowing the CCD to safely acquire the filament beam profile with a lens (*f* = 100 mm)^42^.

Figure 2 presents the experimental and simulated (see Methods) normalized intensity profiles at the geometrical focus, for various initial separation distances between the beams. For the initial separation larger than 1250 μm, we observe negligible deviations from the initial separation (dashed lines in Fig. 2A, B) after 5 meters of propagation indicating that the interaction between the beams is weak for large separation distances.

Between 1250 and 330 μm of initial separation, the attraction between the beams becomes stronger as their separation at the focal point is smaller than the initial separation. The small discrepancy between the simulation and experimental results is due to the fact that, in the experimental case, the final intensity distribution at the focusing plane was averaged over 500 laser shots. On the contrary, in the simulations only one laser shot (without noise) was simulated. Below 330 μm of initial beam separation, the attraction between the two beams results in their fusion into a stable single filament. Figure 2C shows the measured (top row) and simulated (bottom row) transverse profiles at selected initial separations. As the distance between the beams decreases from 1500 μm to 660 μm, their separation reduces after 5 meters of propagation until they fuse into a single entity surrounded by a higher energy reservoir that lacks rotational symmetry in the transverse *x*–*y* plane (see Fig. 2C at 660 μm separation). In the region of stable single filament generation (330 μm – 0 μm), the reservoir has a weaker local intensity but its cross-section is larger with a rotationally symmetric profile in the transverse *x*–*y* plane (see Fig. 2C at 330 and 0 μm separations).

**Figure 3:**
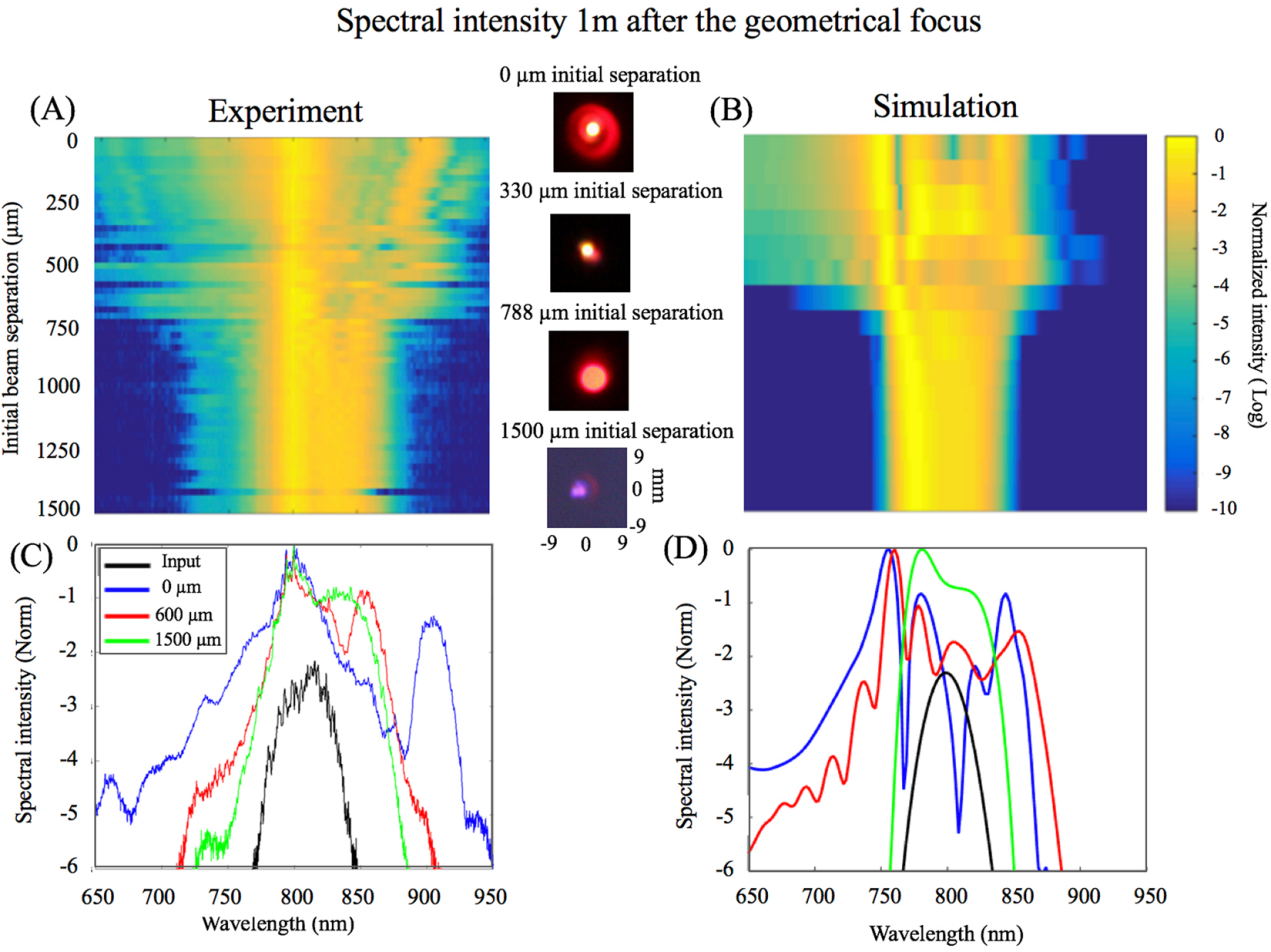
(**A**) Measured and (**B**) calculated spectral intensity of combined beams as a function of initial beam separation. Pictures of the measured conical emission at selected initial beam separations are shown in the middle. (**C**) Measured and (**D**) calculated spectrum for selected initial beam separations compared to the spectrum of the individual sub-critical beams.

Figure 3 shows the spectra measured one meter after the geometrical focus. In the simulations, the intensity of the output spectra are averaged over the whole transverse plane (*x*–*y*) at *z* = 6 m (where the overall spectra were measured experimentally).

Spectral broadening with respect to the input spectrum (black in Fig. 3C, D) is observed even in the regime of negligible attraction (green in Fig. 3C, D). In this regime, the spectral broadening due to the co-propagation of the two beams is negligible when compared to the propagation of any single beam (see Supplementary Fig. 1). When the beams are initially separated by a distance smaller than 850 μm, the spectrum broadens both towards the visible and the infrared. This broad supercontinuum is an indication of the interplay of the non-linear refractive index in both sub-critical beams not seen during propagation of the individual beams. In this situation, the fusion of the two beams, i.e. the transfer of energy is greatest. It is not unreasonable therefore to expect shot-to-shot variations in the position of the attracted centroid.

The transition between regimes of attraction and fusion was considered theoretically by Bergé *et al*.^32, 33^. Using their nomenclature and considering two in-phase beams, a critical separation value, *δ*_*c*_, below which the fusion occurs, can be derived. In our experiments, the beams had a radius of *ρ* = 0.2 mm (measured in the focal plane at *z* = 5 m) and *P*_*1*_ = 0.72*P*_cr_ and *P*_*2*_ = 0.68*P*_cr_. For these parameters, the critical fusion distance *δ*_*c*_ can be calculated as a root of Eq. (14) in ref. 32 and is equal to *δ*_*c*_ ≈ 600 μm. It can be seen from Fig. 3 that the sudden broadening of the spectrum starts at approximately 700 μm and peaks at around 600 μm initial separation, confirming the presence of nonlinearities, commonly seen for a single filament, induced by the beam combination. These observations also demonstrate the ability to control the spectral broadening by varying the separation between the two beams, while at the same time keeping their initial intensity constant and below the critical value for filamentation.

**Figure 4:**
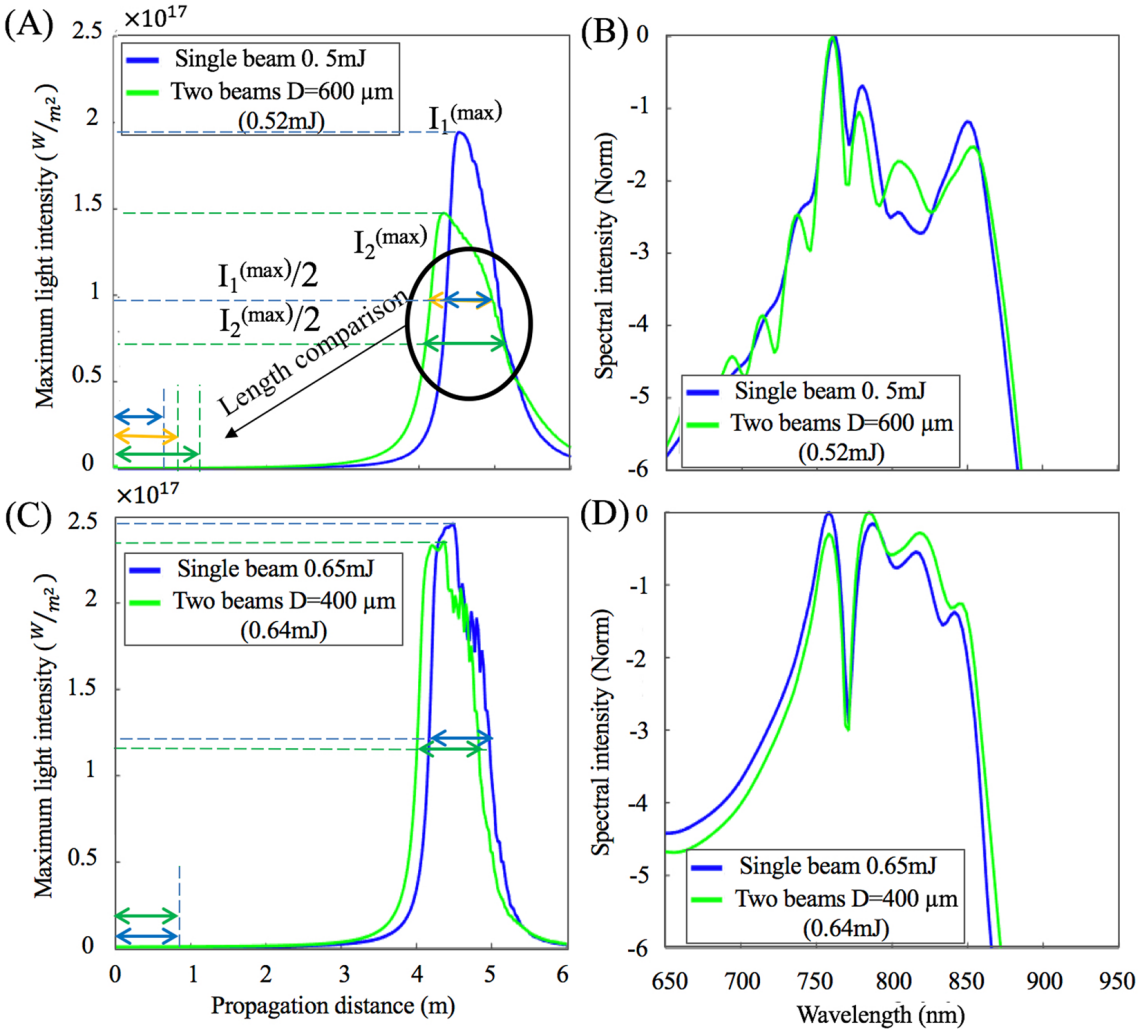
(**A**) and (**C**) calculated intensity along propagation for a single beam (blue) and two combined beams (green) with comparable powers. (**B**) and (**D**) spectral intensity for a single beam (blue) and two combined beams (green) with comparable powers.

Figure 4 compares both the maximum laser light intensity as a function of the propagation distance *z* (Fig. 4A, C) and the normalized spectral intensity at *z* = 6 m (Fig. 4B, D) for a single filament created from a single beam (blue) to that of the filament created by the nonlinear combination of two sub-critical beams (green). The input energy of the single beam is chosen based on the resulting energy of the two interfering input Gaussian beams. As shown earlier, for initial separations larger than 700 μm, the two beams do not fuse, preventing the generation of a filament. As the initial separation decreases below 700 μm, the two beams start to fuse and create a single filament. For instance, with a beam separation of 600 μm (shown in Fig. 4A, B), the total input energy is 0.52 mJ and the filament is created. The maximum light intensity of a filament created by combining the two beams is lower than the intensity of a filament created using a single beam with 0.5 mJ energy, but the filament resulting from the fusion starts earlier and﻿﻿ is longer. Here‚ the length is calculated as the distance over which the light intensity is larger than half of the maximum value, where it results in a similar supercontinuum spectrum 1 m after the geometrical focus (Fig. 4B). For smaller initial separations (e.g. 400 μm shown in Fig. 4C, D), the discrepancy between the two filaments is negligible, regardless of whether they were created from a single pulse of 0.65 mJ or two pulses of 0.32 mJ. The maximum intensity and the length of the filaments are very similar with comparable spectra, even if the filament resulting from fusion of the two beams is created a little earlier.

In summary, we showed the significance of using sub-critical pulses for the creation and control of filaments: two pulses with a total energy equal to or greater than that of a single beam but distributed over two beams separated by a precisely controlled distance are shown to induce identical spectral broadening. We infer from the model, and the close correspondence between the theoretical and experimental results that the starting point and the length of the filament created from the fusion of the two lower powered beams can be controlled by the initial separation between their centroids. Our results open new opportunities for precise engineering of filamentation using low-power laser pulses. Future studies will investigate the dependence of energy transfer between two filaments on time and phase differences in the two parallel sub-critical beams. We view these studies as the next steps towards understanding energy transfer between more complex beam structures involving multiple beams, including beams of different wavelengths.

## Methods

The numerical simulations were performed using a (3 + 1) dimensional split-step Fourier scheme to solve the Nonlinear Schrödinger Equation describing the evolution of the slowly varying envelope of the electric field $E(x,y,z,t)$of ultrashort pulses propagating in air^1^:

1 $$\begin{array}{c}\frac{\partial E}{\partial z}=\frac{i}{2{k}_{0}}(\frac{{\partial }^{2}}{\partial {x}^{2}}+\frac{{\partial }^{2}}{\partial {y}^{2}})E-i\frac{{k}^{\text{'}\text{'}}}{2}\frac{{\partial }^{2}E}{\partial {t}^{2}}+i{k}_{0}{n}_{2} {\mathcal R} (|E{|}^{2},t)\\ \quad \quad \,\times \,E-(\frac{{\sigma }_{B}}{2}+i\frac{{k}_{0}}{2{\rho }_{c}})\rho E-\frac{{\beta }^{(K)}}{2}{|E|}^{2K-2}E,\end{array}$$

Here, *E* is normalized in such a way that $|E{|}^{2}$ represents the light intensity expressed in W/m^2^. The total electric field is given as $\, {\mathcal E} (x,y,z,t)=E(x,y,z,t)\exp (-i{\omega }_{0}t)$, where ${\omega }_{0}=\frac{2\pi c}{{\lambda }_{0}}$ denotes the light angular frequency, $c$ is the speed of light in vacuum, and ${\lambda }_{0}=800$ nm is the central free-space wavelength of the femtosecond pulse. ${k}_{0}=\frac{2\pi }{{\lambda }_{0}}$ is the free space wavenumber, $k^{\prime\prime} =2$ fs^2^/cm ^43^ and ${n}_{2}=5.57\times {10}^{-19}$ cm^2^/W^44^ are the group velocity dispersion and nonlinear index of refraction for air at the wavelength $\,{\lambda }_{0}$, respectively. The inverse Bremsstrahlung cross-section responsible for plasma absorption is denoted by ${\sigma }_{B}=5.47\times {10}^{-20}\,$cm^2^, the critical plasma density at which air becomes transparent is given by ${\rho }_{c}=1.74\times {10}^{21}$ cm^−3^, and the multiphoton absorption coefficient ${\beta }^{(K)}=3.67\times {10}^{-95}$ cm^13^/W^7^, where $K=8$ shows the number of photons needed to be absorbed to overcome the effective ionization potential ionization potential of air ${U}_{i}=11$ eV^43^. The nonlinear response of air is described by

2 $$ {\mathcal R} (|E{|}^{2},t)=(1-\alpha )|E(t){|}^{2}+\frac{\alpha \,({{\rm{\Gamma }}}^{2}+{{\omega }_{R}}^{2})}{{\omega }_{R}}{\int }_{-\infty }^{t}\exp [-{\rm{\Gamma }}(t-\tau )]\sin [{\omega }_{R}(t-\tau )]{|E(\tau )|}^{2}d\tau ,$$

taking into account both the instantaneous Kerr effect and the delayed response due to stimulated molecular Raman scattering with the weights $(1-\alpha )$ and $\,\alpha =0.5$, respectively. The characteristic relaxation time for oxygen molecules is taken as ${\Gamma }^{-1}=70$ fs and the molecular response frequency is given by ${\omega }_{R}=16$ THz. The time evolution of the electron density is governed by equation

3 $$\frac{\partial \rho }{\partial t}={\sigma }_{K}({\rho }_{{\rm{nt}}}-\rho )|E(t){|}^{2K}+\frac{{\sigma }_{B}}{{U}_{i}}\rho |E(t){|}^{2},$$

where ${\sigma }_{K}={\beta }^{(K)}/(K\hslash {\omega }_{0}{\rho }_{{\rm{nt}}})$ is the multiphoton ionization coefficient, $\hslash {\omega }_{0}$ denotes the energy of a single photon at the frequency ${\omega }_{0}$, and ${\rho }_{{\rm{nt}}}=5\times {10}^{18}$ cm^−3^ is the density of neutral oxygen molecules. The numerical values of the parameters for air were taken from ref. 1. Using the values presented here, starting with a $45$ fs Gaussian beam ($4.25$ mm FWHM) focused by a $5$ m lens in air, the critical peak power for filamentation was found to be ${P}_{{\rm{cr}}}=6.3$ GW.

## Electronic supplementary material

Supplementary Figure
