# Supplementary material for: Free-Space Nonlinear Beam Combining for High Intensity Projection
Source: Sci Rep. 2017 Aug 31;7:10147. doi: 10.1038/s41598-017-10565-x (PMC5579033; doi:10.1038/s41598-017-10565-x)
Supplement: Supplementary file 1 — Supplementary Figure [file 41598_2017_10565_MOESM1_ESM.pdf]

# Free-Space Nonlinear Beam Combining for High Intensity Projection

**(Supplementary Information)**

**Shermineh Rostami,** [1] **Wiktor Walasik,** [2] **Daniel Kepler,** [1]

**Matthieu Baudelet,**[1, 3] **Natalia M. Litchinitser,** [2] **and Martin Richardson**[1*]

1 Laser Plasma Laboratory, Townes Laser Institute, College of Optics and Photonics, University of Central Florida, USA

2 Department of Electrical Engineering, University at Buffalo, The State University of New York, Buffalo, New York 14260, USA

3 National Center for Forensic Science /Chemistry department, University of Central Florida, USA

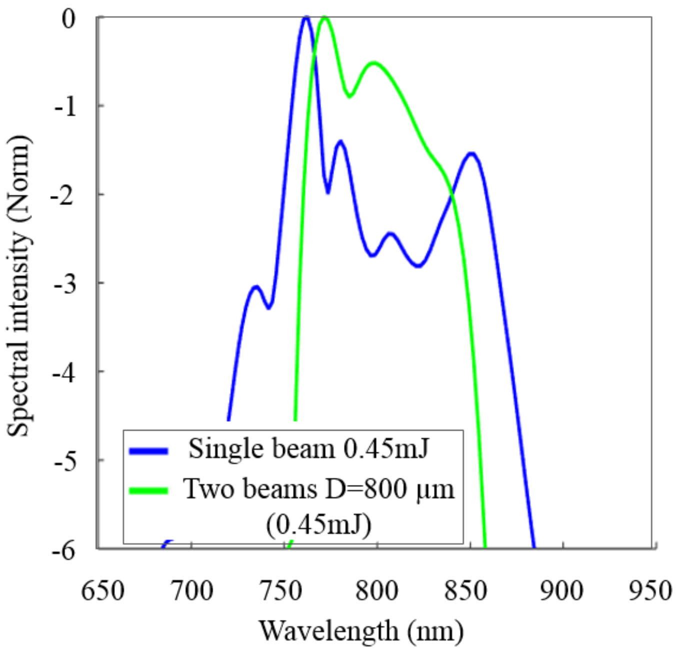


Supplementary Fig.1: Spectral intensity for a single beam (blue) and two combined beams (green) with comparable powers with initial separation of 800 µm.
